# An evaluation of the evolution of the gene structure of dystroglycan

**DOI:** 10.1186/s13104-016-2322-x

**Published:** 2017-01-03

**Authors:** Andrea Brancaccio, Josephine C. Adams

**Affiliations:** 1 Istituto di Chimica del Riconoscimento Molecolare, CNR, Istituto di Biochimica e Biochimica Clinica, Università Cattolica del Sacro Cuore, L.go F. Vito 1, 00168 Rome, Italy; 2 School of Biochemistry, University of Bristol, Biomedical Sciences Building, University Walk, Bristol, BS8 1TD UK

**Keywords:** Dystroglycan, Gene structure, Exon–intron junctions, IG domain, Intron expansion, Metazoan

## Abstract

**Background:**

Dystroglycan (DG) is an adhesion receptor complex composed of two non-covalently associated subunits, transcribed from a single gene. The extracellular α-DG is highly and heterogeneously glycosylated and binds with high affinity to laminins, and the transmembrane β-DG binds intracellular dystrophin. Multiple cellular functions have been proposed for DG, notwithstanding that its role in skeletal muscle appears central as demonstrated by both primary and secondary severe muscular dystrophic phenotypes collectively known as dystroglycanopathies. We recently analysed the molecular phylogeny of the DG core protein and identified the α/β interface, transmembrane and cytoplasmic domains of β-DG as the most conserved region. It was also identified that the IG2_MAT_NU region has been independently duplicated in multiple lineages.

**Results:**

To understand the evolution of dystroglycan in more depth, we investigated dystroglycan gene structure in 35 species representative of the phyla in which dystroglycan has been identified (i.e., all metazoan phyla except Ctenophora). The gene structure of three exons and two introns is remarkably conserved. However, additional lineage-specific introns were identified, which interrupt the coding sequence at distinct points, were identified in multiple metazoan groups, most prominently in ecdysozoans.

**Conclusions:**

A coding DNA sequence (CDS) intron that interrupts the encoding of the IG1 domain is universally conserved and this intron is longer in gnathostomes (jawed vertebrates) than in other metazoans. Lineage-specific gain of additional introns has occurred notably in ecdysozoans, where multiple introns interrupt the large 3′ exon. More limited intron gain has also occurred in placozoa, cnidarians, urochordates and the DG paralogues of lamprey and teleost fish.

**Electronic supplementary material:**

The online version of this article (doi:10.1186/s13104-016-2322-x) contains supplementary material, which is available to authorized users.

## Background

Dystroglycan (DG) is an adhesion receptor complex that provides mechanical stability to a wide variety of cells and tissues in mammals, zebrafish, *Drosophila melanogaster* and *Caenorhabditis elegans*. It forms a bridging element that connects the internal cytoskeleton to basement membrane extracellular matrix [1]. In this regard, the two subunits of dystroglycan, α-DG and β-DG, play different roles. α-DG is highly glycosylated, located extracellularly and binds with high affinity to laminins and other laminin globular (LG) domain-containing proteins and proteoglycans [2]. β-DG spans the plasma membrane and is anchored to the actin-binding protein, dystrophin, thereby forming a direct link to the actin cytoskeleton [3].

DG has been related to the function of skeletal muscle since its initial identification in rabbit sarcolemma [4]. The calcium-dependent, high-affinity binding established between α-DG and laminin is believed to depend mainly on binding between carbohydrate moieties attached to the central elongated mucin-like domain of α-DG and the C-terminal LG domains of laminin α chains [2]. The conditional disruption of the dystroglycan gene in mice causes muscular dystrophy, and severe congenital muscular dystrophy phenotypes emerge when α-DG is hypoglycosylated [5, 6]. Collectively, there is a subgroup of muscular dystrophies currently referred to as dystroglycanopathies, which are classified as (i) primary, when the DG core protein is mutated [7–9], and (ii) secondary, when genetic alterations of glycosyltransferases, or of other proteins important for DG maturation, are involved [10].

**Fig. 1:**
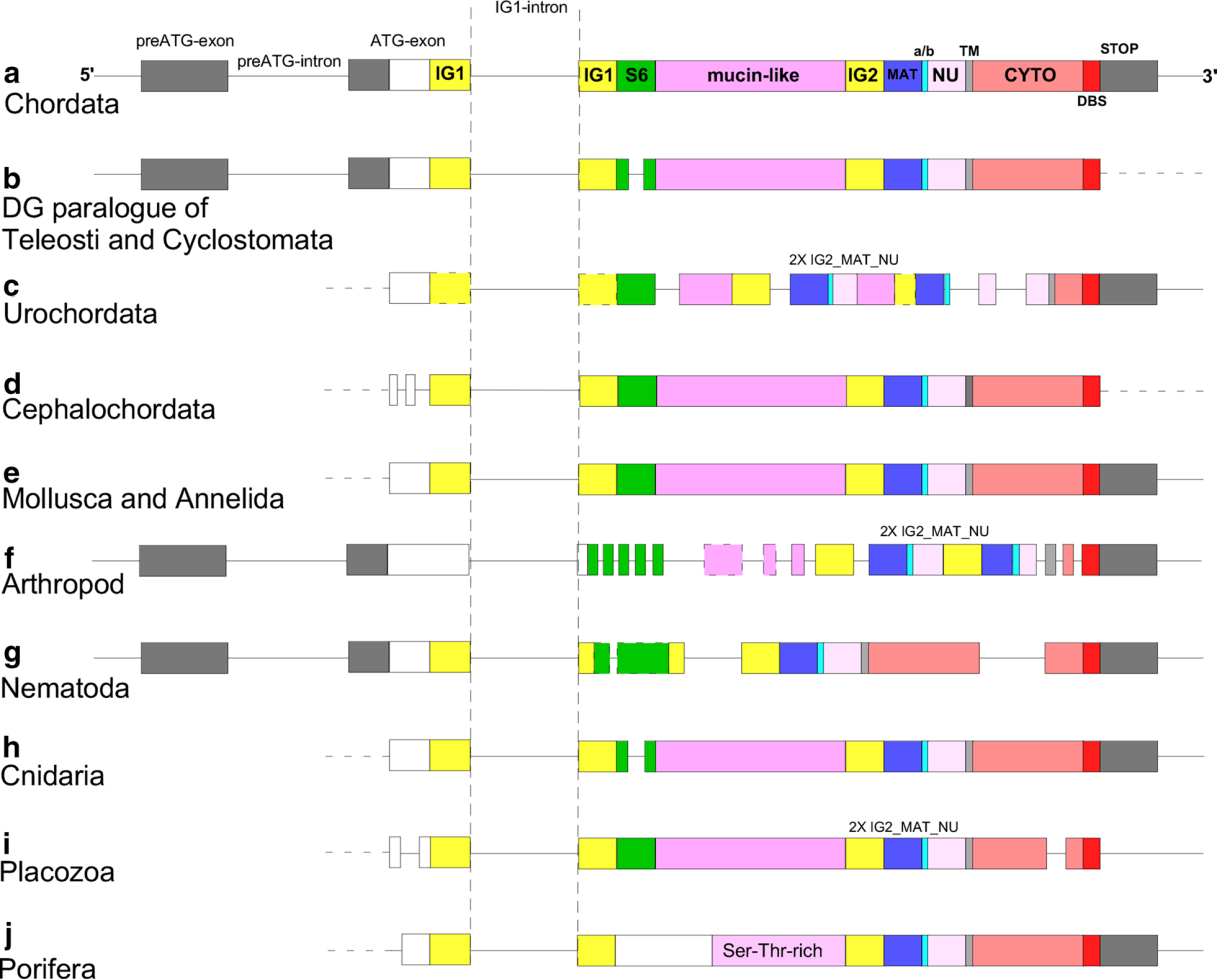
Architecture of dystroglycan genes from different metazoan phyla. **a** The typical organization of the DG gene that is found in most Chordata. This panel also represents the DG gene structure identified in a hemichordate species (*S. kowalevskii*), the echinoderm *S. purpuratus* and the cephalopod mollusc *O. bimaculoides*. **b** Some species of Teleostei and Cyclostomata (Table 2) also have a paralogous *DAG1* gene (designated as *DAG1a*, [26]) with an additional short intron that interrupts the sequence encoding the S6 domain. **c** *Ciona intestinalis* (Urochordata); **d** *Branchiostoma floridae* (Cephalochordata). **e** Typical DG gene organisation of Mollusca (Bivalvia and Gasteropoda) and Annelida; **f** *Drosophila melanogaster* (Arthropoda). **g** *Caenorhabditis elegans* (Nematoda). **h** *Hydra magnipapillata* (Cnidaria). **i** *Trichoplax adhaerens* (Placozoa). **j** *Amphimedon queenslandica* (Porifera). In all diagrams, pre-coding exons are in *grey* and coding exons are in *white* or coloured to represent the encoded protein domains. IG domains of *Ciona* DG show a lower degree of homology. Alternatively spliced exons in *Drosophila* are boxed by *dashed lines*. Introns are indicated by *black lines*. Boundaries of the IG1-intron are indicated by two *vertical dashed lines*. Key: IG1 and IG2, immunoglobulin-like domains; S6, S6-like domain; MAT, C-terminal region of α-dystroglycan upstream of the Gly-Ser maturation site; a/b, α/β DG proteolysis site, NU, natively unfolded region that forms the N-terminal region of the ectodomain of β-dystroglycan; TM, transmembrane; Cyto, cytoplasmic domain; DBS, dystrophin-binding site. Not to scale. Underlying data and accession codes are given in Tables 1 and 2

The two subunits of DG are transcribed from a single gene. The domain organization of the primary protein product is as follows: a signal peptide; immunoglobulin-like domain 1 (IG1); S6 domain (so-called because of its similarity to ribosomal protein S6, [11]; a mucin-like central region; immunoglobulin-like domain 2 (IG2); the so-called “α/β maturation interface” (MAT) which includes a 50 residue region of α-DG after the IG2 domain and the Gly-Ser site of proteolysis; a natively unfolded domain within the ectodomain of β-DG (NU); a single transmembrane domain and a cytoplasmic region that includes the dystrophin-binding site (DBS) at its C-terminus (see Fig. 1) [12]. The IG1 domain of α-dystroglycan (PDB:1U2C) adopts an immunoglobulin-like fold for which twitchin (PDB:1WIT) is the closest structural neighbour [11]. The α-DG IG1 domain is also a very close structural neighbour of the natural cysteine peptidase inhibitor of *Leishmania mexicana* (PDB:2C34) (Z-score of 5.1 and an RMSD of 3.2 Å over 82 residues) [13].

For the maturation of α-DG, the N-terminal region (IG1 and S6 domains) is considered highly important. Indeed, the N-terminal region in isolation displays a residual laminin-binding activity [11] and is likely to be important for directing the actions of a plethora of enzymes required for the glycosylation of α-DG [7, 14]. Based on pioneering recombinant protein analysis, the N-terminal domain of α-DG has been suggested to represent an autonomous module [15]. This module can be liberated by furin-driven proteolysis [16, 17] within the extracellular space and/or body fluids [18–21]. It is also speculated that the IG1 domain might function in self-recognition (in *cis*) of carbohydrate moieties that protrude from the neighbouring mucin-like region, and therefore could have additional functions within the glycosylation and maturation pathway of the dystroglycan precursor molecule [14].

Recently, we conducted the first extensive evolutionary study of the dystroglycan core protein and demonstrated a high degree of conservation in all metazoan phyla except ctenophores, where DG is absent from the two available species, *Mnemiopsis leidyi* and *Pleurobrachia bachei* [12]. Our study demonstrated that the most conserved region of DG encompasses the second IG-like domain (IG2), the α/β interface that is important for establishing non-covalent contacts between the two subunits, the ectodomain of β-DG (the MAT_NU module that includes the Gly-Ser α/β maturation site) and the transmembrane and cytoplasmic domains [12]. A major unexpected finding was that multiple, presumably independent, lineage-specific duplication/domain shuffling events have led to repetitions of the IG2_MAT_NU module in species of hemichordates (2X), arthropods (2X), placozoa (2X) and in particular in the cnidarian sea anemone *Nematostella vectensis* (6X).

Apart from information on the DG gene in a few mammalian species [22, 23] or on the alternative spliced variants of *Drosophila melanogaster* [24], no detailed investigation of the gene organization of dystroglycans has been conducted. Here, we have investigated the evolution of the dystroglycan gene with reference to the metazoan phyla previously identified to encode DG [12]. Especially, we were interested to study: (i) the overall degree of conservation of exon–intron organization of the dystroglycan (DG) gene; (ii) the relationship between DG domain organization and exon structure, particularly with regard to the IG_MAT_NU domain duplications identified previously in certain phyla, and (iii) if distinctions at the level of exon/intron organization have emerged by divergence in specific lineages.

## Results

### Dystroglycan gene structure is remarkably conserved

Table 1 reports the details of DG gene organization with reference to 35 metazoan species that represent the major metazoan phyla which we previously identified to encode DG [12]. These prior studies did not identify DG in Ctenophora [12]. The identified DG gene organisations are schematized in Fig. 1, which also indicates the disposition of the encoded protein domains between the exons. It is apparent that DG gene structure is simple in all chordate species analysed to date (Fig. 1a), also in bivalve and gastropod molluscs and annelids (Fig. 1e). In all these species, the DG gene includes a single intron within its coding DNA sequence (CDS). This intron interrupts the DNA sequence encoding the IG1 domain and we therefore refer to it as the IG1-intron. Our survey demonstrates that an intron at this position is universally present (Fig. 1), albeit with a variable size (Table 1 and see section below). In Chordata, Cephalopoda, Arthropoda and Nematoda, the ATG-containing exon that anticipates the IG1-intron is preceded by an additional large (40–60 kb in mammals; Table 1) intron (designated pre-ATG intron in Fig. 1). The DG genes of these species also include a relatively short (ranging from 89 to 595 bp) non-coding exon, designated here the pre-ATG exon. This non-coding exon was not identified in the DG genes of urochordate, cephalochordate, bivalve and gastropod molluscs, or in DG genes of species representative of Annelida, Placozoa or Porifera (Fig. 1), however a pre-ATG exon appears present in the DG gene of the cnidarian sea anemone *Exaiptasia pallida* (AIPGENE266, [26]). In view that this exon is 5′ to the ATG codon, the possibility that the pre-ATG exon is not recognizable in other non-bilaterian species due to incomplete or inaccurate genome annotations cannot be ruled out at this time. Indeed, in our previous analysis of the DG of the sponge, *Oscarella carmela,* the predicted protein sequence identified was incomplete at the N-terminus [12]. We subsequently identified a complete predicted DG sequence in the demosponge *Amphimedon queenslandica* (Table 1). Strikingly, the DG gene of *A. queenslandica*, in common with those of deuterostomes, most molluscs and annelids, contains the entire protein coding sequence in only two exons (Fig. 1j). Although partial DG sequences can be identified in other sponges, e.g. *O. carmela* [12], it was not possible to identify an intact 5′UTR in these species. Thus, at present it is not possible to establish if the *A. queenslandica* DG gene structure is unique or representative of other sponges. In addition, we cannot rule out the possibility that alternative splicing takes place at the 5′ end of the DG gene in these species.

**Table 1:** Summary of DG gene structures from species representative of all the animal groups in which a DG gene was identified

Metazoan main taxa *Species*	Exon1 (preATG exon) (bp)	Intron1 (preATG intron) (bp)	Exon2 (ATG exon) (bp)	Intron2 (IG1-intron) (bp)	Exon3 (stop codon & 3′UTR) (bp)	Gene accession code	Code name	Notes
Chordata (mammals*)*								
*H. sapiens*	302	39,985	401	19,977	4819	ENSG00000173402	>Hs	
*M. musculus*	139	45,369	398	8351	3707	ENSMUSG00000039952	>Mm	
*C.l.familiaris*	375^d^	48,846	383	16,520	4968	ENSCAFG00000011207	>Clf	1 add. preATG exon–intr.
*O. anatinus*	161	58,270	398	30,785	5789	ENSOANG00000013307	>Oa	
*D. novemcinctus*	203	62,147	400	22,513	2403^a^	ENSDNOG00000016917	>Dn	
Chordata (aves)								
*G. gallus*	^h^	^h^	317	8464	2409	ENSGALG00000027710	>Gg	
*F. albicollis*	175	34,691	410	12,789	2740	ENSFALG00000009006	>Fa	
Chordata (reptiles)								
*A. carolinensis*	392	56,285	403	26,079	7196	ENSACAG00000007264	>Ac	
*P. sinensis*	^h^	^h^	380	25,784	6793	ENSPSIG00000016851	>Ps	
Chordata (amphibia)								
*X. tropicalis*	125	19,428	354	8686	2917	ENSXETG00000005928	>Xt	
Chordata (teleostei)								
*D. rerio*	169	39,769	528	14,466	2899	ENSDARG00000016153	>Dr	
*G. morhua*	^h^	^h^	345	1126	2310^a^	ENSGMOG00000017787	>Gm1	
*X. maculatus*	595	8826	566	2800	3850	ENSXMAG00000015708	>Xm1	
*T. rubripes*	^h^	^h^	366	818	2307^a^	ENSTRUG00000007345	>Tr1	
Chordata (chondrichthyes)								
*C. milii*	^h^	^h^	276^b^	11,246	2373^a^	KI635869.1 (Elephant Shark Genome Project@IMCB)	>Cmi	
Chordata (cyclostomata)								
*P. marinus*	172	24,308	399	1218	2218^a^	ENSPMAG00000000367	>Pm1	
Urochordata								
*C. intestinalis*	^h^	^h^	348	6249	3703	gene 293,437 (Metazome)	>Ci	4 add. 3′ introns
Cephalochordata								
*B. floridae*	^h^	^h^	708^b^	10,023	1653^a^	fgenesh2_pg.scaffold_27000085 (Metazome)	>Bf	2 add. large 5′ introns
Hemichordata								
*S. kowalevskii*	161	5069	309	7194	5506	Sakowv30014893 m.g (Metazome)	>Sk	
Echinodermata								
*S. purpuratus*	^h^	^h^	300	8426	2373^a^	LOC581503 (Metazome)	>Spu	
Arthropoda (insecta)								
*D. melanogaster*	437	8235	322	284^e^	5774	FBgn0034072 (EnsemblMetazoa)	>Dm	12 add. 3′ introns
*T. castaneum*	^h^	^h^	192^b^	46	3153^a^	LOC663372 (Metazome)	>Tc	4 add. 3′ introns
Arthropoda (crustacea)								
*D. pulex*	^h^	^h^	723	341	2956	DAPPUDRAFT 300,674 (EnsemblMetazoa)	>Dpu	3 add. 3′ introns
Arthropoda (chelicerata)								
*I. scapularis*	^h^	^h^	111^b^	7852	2397^a^ (2796)^a,f^	ISCW015049 (EnsemblMetazoa)	>Is	2 add. 3′ introns^g^
Mollusca (cephalopoda)								
*O. bimaculoides*	561	14,288	1383	8418	2310^a^	Ocbimv22032669 m.g (Metazome)	>Ob	
Mollusca (bivalvia)								
*C. gigas*	^h^	^h^	231	3133	2286^a^	CGI_10020032 (EnsemblMetazoa)	>Cg	
Mollusca (gastropoda)								
*L. gigantea*	^h^	^h^	137^c^	3043	2185^a^	LgGsHFWreduced.7288 (Metazome)	>Lg	
Annelida (sedentaria)								
*C. teleta*	^h^	^h^	210	48	3408	CapteG183589 (Ensembl/Metazoa)	>Ct	
Annelida (clitellata)								
*H. robusta*	^h^	^h^	186	276	2602	HelroG188507 (EnsemblMetazoa)	>Hr	
Nematode (chromadorea)								
*C. elegans*	89	5376	229	328	2236	WBGene00000961 (Metazome)	>Ce	3 add. 3′ introns
Nematode (secernentea)								
*C. remanei*	^h^	^h^	216	319	1653	CRE07443 (EnsemblMetazoa)	>Cr	3 add. 3′ introns
Cnidaria (hydrozoa)								
*H. magnipapillata*	^h^	^h^	210^b^	1751	2189	Hydra_232607 (Metazome)	>Hm	1 add. 3′ S6 intron
Cnidaria (anthozoa)								
*N. vectensis*	^h^	^h^	162^b^	2567	5553^a^	estExt_fgenesh1_pg.C_1310045 (JGI)	>Nv	1 add. 3′ S6 intron
Placozoa								
*T. adhaerens*	^h^	^h^	114^b^	320	2559^a^	TriadG60041 (EnsemblMetazoa)	>Ta	1 add. 5′ and 1 add. 3′introns
Porifera (demospongiae)								
*A.queenslandica*	^h^	^h^	170^b^	47	4254^a^	Aqu1.217766 (EnsemblMetazoa)	>Aq	

For species in which additional introns are present, either upstream (5′) or downstream (3′) of the IG1-intron (intron2), these introns are reported in the Notes column. In these species, the value reported for E1 and/or E3 refers to the combined size originating from all the resulting exons

^a^The genome-annotated sequence ends at the stop codon

^b^Additional nucleotides 5′ to the initial ATG codon may be missing

^c^The annotated gene sequence starts slightly downstream of the ATG codon

^d^An additional pre-ATG exon is reported > 100 Kb upstream

^e^Due to divergence, *D. melanogaster* DG lacks an IG1 domain however the IG1-intron is located in a similar 5′ position to other species that contain the IG1 domain

^f^A recent study has demonstrated that a gene region that was previously considered to code for an intronic sequence is an exon, giving rise to a predicted protein product of 968 aa instead of 835 aa [25]

^g^The first additional intron is also present within the IG1 domain

^h^Not present or not annotated. See Fig. 1 for schematic details

### Evidence for dystroglycan gene duplications and intron gain in some metazoans

In some species of teleost fish, such as *Takifugu rubripes*, the presence of a duplication event involving the *Dag1* gene has been established [27]. We identified two additional bony fish species with two *Dag1* paralogues (Table 2). In line with the designations of Pavoni et al., we designated as *Dag1a* the paralogue that contains an additional short (126–726 bp) intron (mini-intron) that interrupts the encoding of the S6 domain of α-DG (Fig. 1b) [27]. Interestingly, *Petromyzon marinus* (Cyclostomata) also has two DG genes, each with similar gene structure to the *Dag1* genes of bony fish (Table 2) (Fig. 1a, b). A short intron that interrupts the S6 domain encoding region is also present in a very similar location in the DG genes of nematodes (*C. elegans*, Fig. 1g) and cnidarians [*Hydra magnipapillata*, Fig. 1h, and *Nematostella vectensis* (Table 1)]. Based on knowledge of the secondary and tertiary structure of the S6 domain of mouse DG, we determined that the mini-intron insertion site is predicted to fall within a loop that connects the antiparallel β3 and β4 strands, and thus lies in the middle of the “floor” of the S6 domain. Thus, the insertion site is not in register with the tertiary structure of the S6 domain (data not shown, further details can be found in [11]).

**Table 2:** Details of the gene structures of the paralogous form of *DAG1* (*DAG1a*) present in species of teleostei and cyclostomata

Species	Exon1 (Pre-ATG exon) (bp)	Intron1 (pre-ATG intron) (bp)	Exon2 (ATG exon) (bp)	Intron2 (IG1-intron) (bp)	Exon3 (bp)	Intron3 (S6 mini-intron) (bp)	Exon4 (includes stop codon) (bp)	Gene accession code	Code name & notes
Chordata (teleostei)									
*G. morhua*	^b^	^b^	^b^	^b^	480	384	1824^a^	ENSGMOG00000003333	>Gm2
*X. maculatus*	^b^	^b^	210	9155	459	726	1806^a^	ENSXMAG00000012250	>Xm2
*T. rubripes*	^b^	^b^	336	2795	403	137	1827^a^	ENSTRUG00000002580	>Tr2 1 add. 5′ intron (?)
Chordata (cyclostomata)									
*P. marinus*	^b^	^b^	159	2684	975	126^c^	1356^a^	ENSPMAG00000009628	>Pm2

^a^The genome-predicted sequence ends at the stop codon

^b^Not present or not annotated (see Fig. 1b for schematic details)

^c^A mini-intron is not present in S6 but within the mucin-like region

Additional introns that interrupt the coding exons at distinct points are present in representatives of some phyla; these appear to correspond to independent, lineage-specific, intron gain events. Specifically, in the DG gene of *Ciona intestinalis* (urochordate) four additional introns (i.e., in addition to the IG1-intron) split the CDS (Fig. 1c). In the DG gene of *Branchiostoma floridae*, introns interrupt the N-terminal coding sequence (Fig. 1d). The DG gene of the arthropod *D. melanogaster* (Fig. 1f), is particularly conspicuous for having acquired a large number of introns. In general, the location of these introns is not in register with the domain organization or domain boundaries of the DG protein [12]. A notable exception is the “mucin-module” that appears to be encoded by the alternatively spliced exons 8 and 9 in the DG gene of *D. melanogaster* (Fig. 1f; [24]). Additional introns are also present in the DG genes of other insects (e.g., *Tribolium castaneum*) and in species representative of other arthropod classes (Crustacea and Chelicerata) (Table 1). The DG gene of *C. elegans* (nematode) includes multiple introns that interrupt the region encoding the IG2 domain and the cytoplasmic domain, respectively (Fig. 1g). Additional introns in the DG gene of *Trichoplax adhaerens* (placozoan) interrupt the N-terminal encoding sequences in a position similar to the additional introns in the DG gene of *B. floridae* (Fig. 1d) and the cytoplasmic domain-encoding region (Fig. 1i). Whereas the 5′ non-coding exon is apparent in many lineages of bilaterians (Fig. 1a, b, f, g), this exon could not be examined in the available species of cnidarians, placozoan, or the sponge *A. queenslandica*, due to uncertain annotation of DG gene structure 5′ to the ATG codon (Fig. 1h, i). Although this phylogenetic overview made evident the extent of conservation of the large 3′ exon of DG genes, the occurrence of additional introns in multiple lineages also makes apparent that few coding regions have been “privileged” from intron gain. However, in the MAT_NU encoding region around the α/β dystroglycan cleavage site intron addition has occurred only in the urochordate lineage (Fig. 1c). It can be noted that the DG protein sequence of urochordates is exceptionally divergent from that of other metazoans [12].

In our prior study of the molecular phylogeny of DG protein, we identified that the region including the IG2_MAT_NU domains has been independently duplicated in a number of phyla (Hemichordata, Arthropoda, Placozoa, Cnidaria). This phenomenon is particularly striking in the DG of the sea anemone *Nematostella vectensis* (Cnidaria) in which six repetitions of IG2_MAT_NU are present [12]. The current study of dystroglycan gene organization makes it clear that these repetitions are not based on duplication of any exon module. In other words, the additional IG2_MAT_NU protein module(s) are never encoded by a unique exon.

### The IG1-intron has undergone lineage-specific expansion during metazoan evolution

**Fig. 2:**
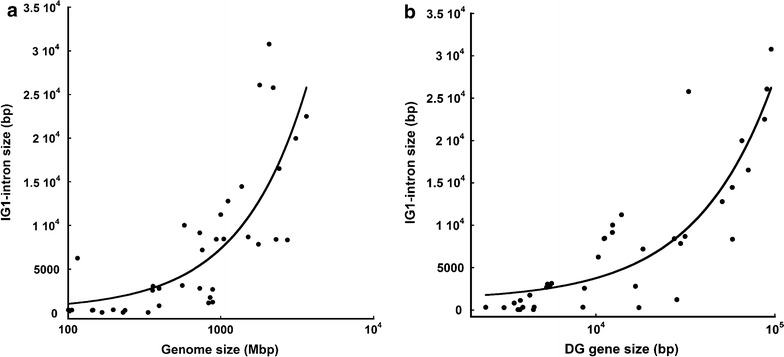
Expansion of the IG1-intron. **a** IG1-intron size as a function of genome size. **b** IG1-intron size as a function of DG gene size. The plots include data from 35 species representative of the metazoan phyla that encode DG. The *fitted lines* in the semi-logarithmic plots in panels **a** and **b** were obtained using a linear equation; the corresponding R^2^ value are 0.68 and 0.75, respectively

In addition to identifying the universality of the IG1-intron, the phylogenetic comparison of DG gene organization highlighted a striking variation in the length of the IG1-intron. A major overall increase in the size of this intron is apparent throughout metazoan evolution: the intron has a minimal size of 47 bp in *A. queenslandica* (Porifera) and 48 bp in *Capitella teleta* (Annelida) and yet comprises up to ~30 kb in *Ornithorhynchus anatinus* and other mammals (Table 1). Indeed, the IG1-intron size was found to increase proportionally with genome size (Fig. 2a) and IG1-intron size also increases with the apparent overall size of *DAG1* gene (Fig. 2b). The underlying numerical data are presented in Additional file 1: Figure S1. To date, no additional CDS have been identified within the IG1-intron.

### Analysis of IG1-intron boundaries

**Fig. 3:**
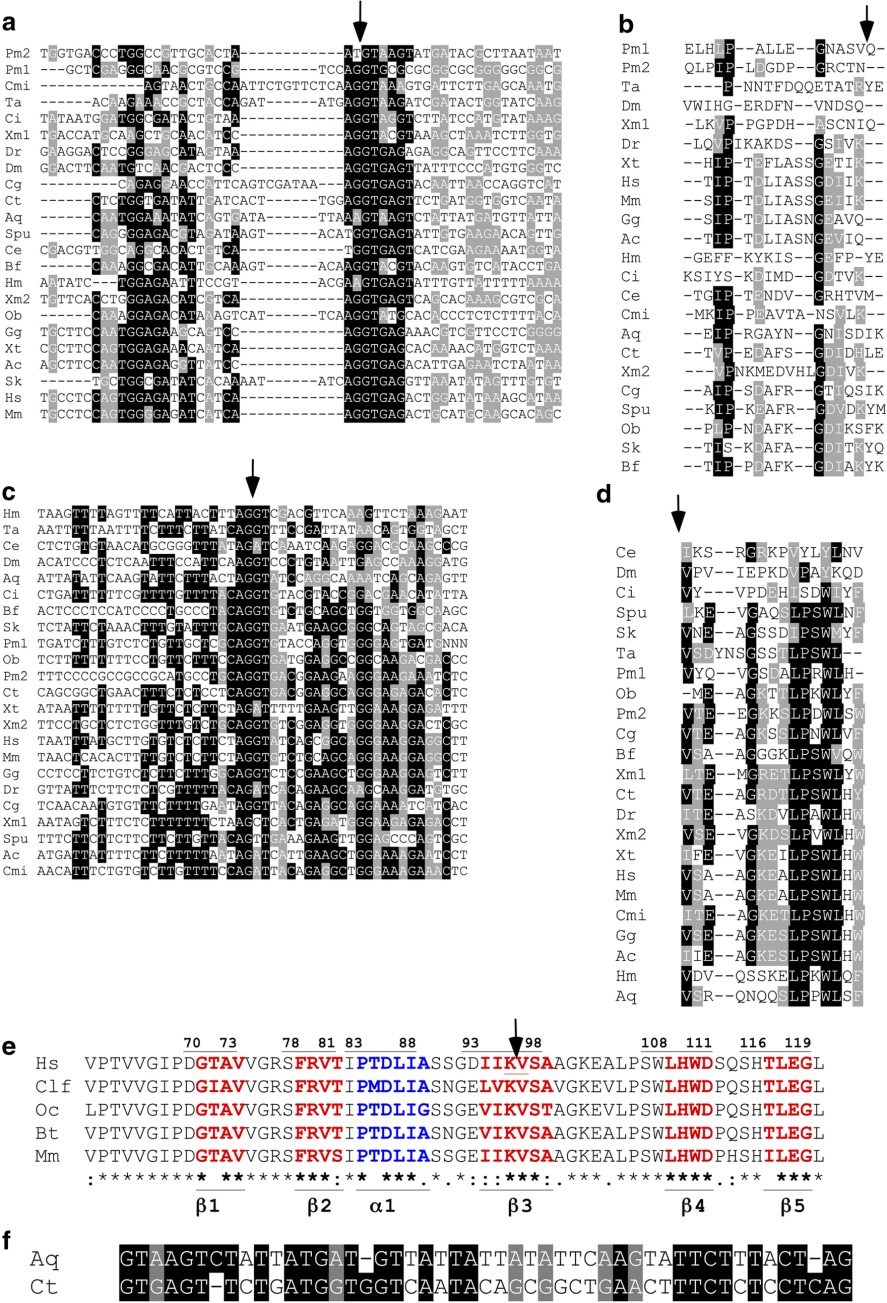
Sequence features of the IG1-intron at the nucleotide and protein levels. **a**, **c** MUSCLE alignment of 50 nucleotides that span the AGGT exon–intron (**a**), or the intron–exon (**c**), boundaries of the IG1-intron. Data are from 23 species representative of the metazoan phyla that encode DG. **b**, **d** Multiple sequence alignments prepared in MUSCLE 3.8 of 15 aa long regions from the IG1 domain that flank the exon–intron insertion site (**b**), or the intron–exon site (**d**) in the same species. The region shown in **b** includes a.a. 81–95 of human DG; the region shown in **d** includes a.a. 96–110 of human DG. **e** The secondary structural elements of the IG1 domain that encompass the intron insertion site (^95^KV^96^ in human DG, *underlined*) [11] demonstrate that the intronic sequence is not in register with the structural organization of the domain. **f** MUSCLE sequence alignment of the IG1-intron sequences from *A. queenslandica* and *C. tellata*. In all alignments, *black background* indicates identical nucleotides or residues in >50% of the sequences, *grey background* indicates conservative substitutions, and a *white background* indicates that the position is conserved in <50% of the sequences. Code names are as in Table 1 with the exception of Oc for *Oryctolagus cuniculus* and Bt for *Bos taurus* in (**e**)

Further investigations of the IG1-intron were initiated by multiple sequence alignment of 50 nucleotides spanning either the exon/intron or intron/exon boundaries of the IG1-intron, using 23 DG gene sequences from representative dystroglycan-encoding species. The alignments demonstrate that the “AGGT exon–intron rule” [28] is largely respected (Fig. 3a, c). However, there are some relevant exceptions, namely *P. marinus, Strongylocentrotus purpuratus*, *C. elegans*, *H. magnipapillata* and *A. queenslandica* for the exon–intron boundary (Fig. 3a) and *Anolis carolinensis*, *Xenopus tropicalis*, *Danio rerio*, *Xiphophorus maculatus*, *Callorhinchus milii*, *S. purpuratus* and *C. elegans* for the intron–exon boundary (Fig. 3c).

The IG1-intron interrupts the coding sequence of human DG between amino acid positions 95 and 96 [22]. MUSCLE multiple sequence alignment of DG protein sequences from the same 23 species and inspection of the locus of IG1-intron splice sites demonstrated that the location of the IG1-intron boundaries is conserved (Fig. 3b, d). However, the intron location does not correspond with the protein domain structure or the IG1 domain boundaries; the IG1-intron interrupts the sequence encoding the middle of the third β-strand of the IG1 domain [11, 14] (Fig. 3e).

As noted above, the smallest IG1-introns are found in *A. queenslandica* (sponge), in *C. teleta* (annelid) and in *T. castaneum* (insect) (Table 1). The IG1-intron sequences of *A. queenslandica* and *C. teleta* are well conserved (Fig. 3f). To the best of our knowledge, no distinct features of the IG1-intron sequence have been identified to date.

## Discussion

### Conservation and diversification of DG gene structure

Collectively, these data demonstrate that the structure of the dystroglycan gene is highly conserved across many metazoan groups and features a universally conserved intron, designated here the IG1-intron, within the 5′ portion of the gene. In some phyla, multiple independent intron gain events have occurred, and gene duplication events have occurred in some teleost fish and in Cyclostomata. Although multiple intron gain seems to be typical of the dystroglycan gene, to the best of our knowledge, this is not the case for several gene families. For example, the RpL14 gene of *D. melanogaster* has fewer introns than the human gene [29]. In the alpha-amylase family of genes, both intron gains and losses have been observed in Bilateria [30].

In *Dag1a* of some teleosts and the DG genes of insects, nematodes and cnidarians, one or more additional introns interrupt the region encoding the S6 domain. Although the IG2_MAT_NU domain region has been duplicated in species from various phyla [12], we did not identify any correlation between the protein domain organization of dystroglycan and its exon/intron structure. With the exception of urochordates, intron gain has not occurred in the MAT_NU region around the α/β processing site.

In vertebrates, a striking characteristic of *Dag1* is its uncomplicated exon/intron arrangement and the presence of only two, relatively large (>15 kb) introns. These two introns are located within the 5′ portion of the gene, making the 3′ region essentially intron-less (Fig. 1). This gene structure is conserved across chordates, whereas the DG gene of species of urochordates (*Ciona intestinalis)*, arthropods (in particular *Drosophila melanogaster)* and nematodes (e.g., *C. elegans*) includes multiple introns. Larger genomes generally contain genes with longer introns [31] and indeed the IG1-intron size increases with genome size (Fig. 2). General studies of eukaryote genomes have indicated a prevalence of intron gain over intron loss; however, in general, apparently very few, if any, introns were gained during the last ∼100 million years of animal and plant evolution [32]. A tendency for extensive intron loss at the 3′ ends of genes has been observed in the genomes of unicellular eukaryotes [33, 34]. The acquisition of additional intronic sequences is considered to possibly represent a mechanism by which novel splice variants can be important for tuning of gene function to particular developmental stages and/or tissue types [35]. Interestingly, rapidly regulated genes are commonly intron-poor [36]. However, it is also the case that dystroglycan has a complex post-translational maturation process in which pre- and post-transcriptional control steps, including intron splicing, are not likely to represent rate-limiting steps [37].

We found that the observed repetitions of the intronless IG2_MAT_NU module in some species [12] do not involve intron sequences, thus all of its tandem repetitions, are found within the large 3′ exon of the DG gene. In general terms, there is extreme variability in the relationship between exon/intron boundaries and the boundaries of protein domain/modules. In some cases, single protein domains are encoded by exons but there are also many examples where a single domain is interrupted by intron(s) [38]. Although there is no significant amino acid sequence homology between DG and these other proteins, it is interesting that a similar exon/intron arrangement as found in the IG1 domain of DG is present in some IG-domain-containing cell-surface receptors, for example, CD4, CD3δ, or NCAM [39].

The biological significance of the IG1 domain for DG function has been underscored by the recent identification of two novel compound heterozygous DG missense mutations, V74I and D111N, that are associated in a patient with asymptomatic hyperCKemia and hypoglycosylation of α-dystroglycan [8]. The mutation T192M, within the β1 strand of the neighbouring S6 domain, also causes hypoglycosylation of α-DG with consequent neuromuscular and brain phenotypes [7]. In view that the IG1 and S6 domains belong to an autonomous globular structural unit at the N-terminus of α-DG [15], the N-terminal region of DG is believed to play some, as yet, unidentified autonomous function both extracellularly and/or intracellularly [18–21].

Further work will be needed to analyse the 5′ and 3′ untranslated regions (UTR) of dystroglycan genes for possible conserved transcription factor binding sites and/or other regulatory elements such as miRNA hybridization sites [23, 40]. A preliminary search shows that organ-specific miRNA target sequences identified in the 3′ UTR of *D. melanogaster* DG (miR9a (CCAAAGA) in myotendinous junction and miR310 s (UGCAAUA) in the brain) [41, 42] are found exactly or with minimal variation (1 nucleotide out of 7) in the dystroglycan mRNA of *Homo sapiens* (5′CCAGAGA and 5′UGCAAUA, respectively), *Mus musculus* (5′CUAAAGA and 5′UGCAAUA, respectively) and *Hydra magnipapillata* (5′CAAAAGA, miR9a-like). This conservation might indicate that some of the regulatory mechanisms observed in *Drosophila melanogaster* might also be relevant to other species.

### A summary model for evolution of the dystroglycan gene

**Fig. 4:**
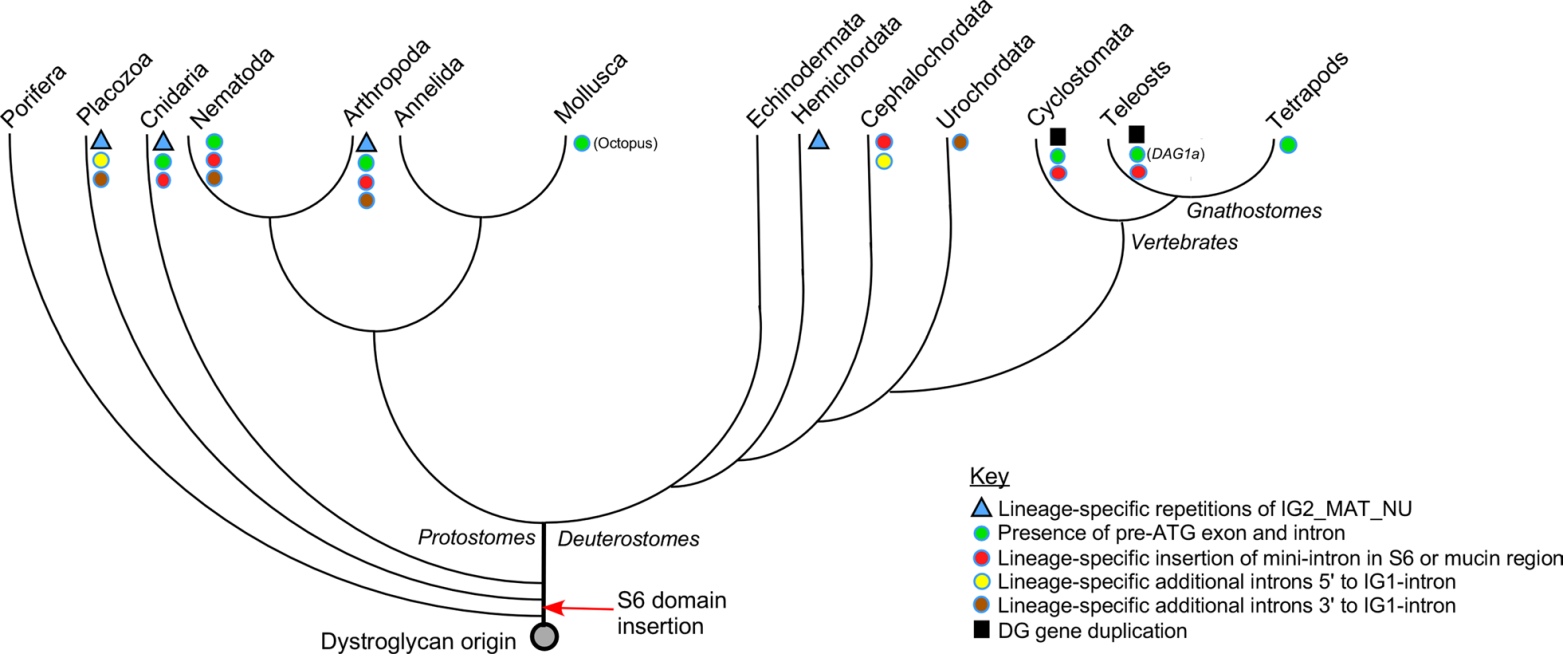
Model of DG gene evolution. The diagram does not include ctenophores due to their uncertain evolutionary placement and that no DG-encoding sequences have been identified in ctenophores. See text for discussion

Figure 4 presents a model of evolutionary changes in the DG gene as identified from our study. This model focuses on the phyla in which DG has been identified to be present, as established from genome-predicted protein sequences and the existence of corresponding mRNA transcripts [12]. DG is not encoded in two species of ctenophores (*Pleurobrachia bachei* and *Mnemiopsis leidyi*), and phylum Ctenophora is not included in the model. Our previous study of the molecular phylogeny of the DG protein demonstrated that the IG2_MAT_NU region and the domains of β-dystroglycan are the most highly conserved regions that might reflect the ancestral form of DG [12]. The current information on *A. queenslandica* DG indicates that the IG1 domain and IG1-intron have been part of the DG gene from its earliest origin. In contrast, the S6 domain appears to have been gained in the last common ancestor of placozoans, cnidarians and bilaterians, perhaps by exon shuffling (Fig. 4). The simple structure of the *A. queenslandica* DG gene, that is highly comparable to the DG gene structures found in annelids, molluscs, cephalochordate and jawed vertebrates, implicates that this gene organisation is likely to reflect the ancestral gene structure.

Given the different number, sizes and positions of additional introns evident in the DG genes of some metazoan groups, it is reasonable to hypothesize that these introns were acquired independently, as lineage-specific evolutionary events [32, 43, 44]. In particular, because multiple additional introns are present in the DG genes of nematodes and arthropods (the major phyla of the Ecdysozoa), but not in annelids or molluscs (the major phyla of the Lophotrochozoa) it can be proposed that intron gain occurred in the last ecdysozoan common ancestor, followed by phylum-specific intron gains or losses in nematodes and arthropods. The current data implicate that the pre-ATG exon was already present in the last bilaterian common ancestor (Figs. 1, 4). However, in view that some of the DG genes analysed in basal metazoans may be incompletely annotated at the 5′ end, this interpretation must be provisional at this time.

Other intron gain events, such as the mini-introns within the S6 or mucin domains, or introns 3′ to the S6 domain, appear to be entirely taxa- or lineage-specific and thus are proposed to be of later evolutionary origin (Fig. 4). Although the organisation of the paralogous *DAG1a* and *DAG1b*, respectively, are similar in lamprey and bony fish, it remains controversial whether the genome-wide duplications that took place in the early vertebrate lineage occurred before, or after, the divergence of cyclostomes, especially in view of the presence of independent gene losses and gains in extant lampreys [45–47].

In conclusion, although a simple organisation of the DG gene with 2 coding exons/1 CDS intron, has been conserved robustly, significant divergence and intron gain has occurred in Ecdysozoa and Urochordata, and to a lesser extent in the placozoan *T. adhaerens*. Generally the newly gained exon/intron architectures are unrelated to protein domain boundaries. In particular, the duplication of IG2_MAT_NU regions that has been identified in species from Urochordate, Arthropoda, Cnidaria and Placozoa is not related to the intron–exon organisation of these DG genes. Further analyses will be needed to investigate whether these aspects of DG gene structure are relevant to genes encoding other cell adhesion molecules.

## Methods

### Identification of DG gene sequences throughout the metazoa

All the gene sequences investigated were retrieved either from the Ensembl database [48] or from the Metazome v3.0 database from the University of California (http://www.metazome.net). Searches were completed as of the end of January 2016. Searches with protein sequences were performed by BLASTP at NCBI Genbank at default parameters and were based on the protein sequences studied in [12]. Dystroglycan gene sequences identified were further confirmed by multiple sequence alignments in MUSCLE 3.8 using the human dystroglycan sequence as a reference. The accepted borders of the relevant dystroglycan domains were taken as described in [12].

### Multiple sequence alignment

Multiple sequence alignments of nucleotide or protein sequences were constructed in MUSCLE 3.8 [49] via the resources of EMBL/EBI (http://www.ebi.ac.uk/Tools/msa) and are presented in BoxShade 3.21 (http://www.ch.embnet.org/software/BOX_form.html). Secondary structure elements are reproduced from PDB 1U2C [11].

### Graphs

Graph presentations and fitted lines were generated using KaleidaGraph (Synergy Software).

## Additional file

**Additional file 1: Figure S1.** Additional figure.

## Abbreviations

CDS: coding DNA sequence
DBS: dystrophin-binding site
DG: dystroglycan
LG: laminin G domain
