# Supplementary material for: An evaluation of the evolution of the gene structure of dystroglycan
Source: BMC Res Notes. 2017 Jan 3;10:19. doi: 10.1186/s13104-016-2322-x (PMC5216574; doi:10.1186/s13104-016-2322-x)
Supplement: Supplementary file 1 — Additional file 1: Figure S1. Additional figure. [file 13104_2016_2322_MOESM1_ESM.docx]

**Animal group and species** **IG1-intron size (bp) Genome Size (Mb)***

Porifera *Amphimedon queenslandica* 47 166.679601

Placozoa *Trichoplax adhaerens* 320 105.631681

Cnidaria *Hydra magnipapillata* 1751 852.2 (Metazome)

Cnidaria *Nematostella vectensis* 2567 356.613585 (Ens/JGI)

Nematoda *Caenorhabditis remanei* 319 145.442736

Nematoda *Caenorhabditis elegans* 328 100.286401

Annelida *Capitella teleta* 48 333.283208

Annelida *Helobdella robusta* 276 235.376169

Mollusca *Lottia gigantea* 3043 359.505668

Mollusca *Crassostrea gigas* 3133 557.717710

Mollusca *Octopus bimaculoides* 8418 2300 (Metazome)

Arthropoda*Drosophila melanogaster* 284 143.725995

Arthropoda *Tribolium Castaneum* 46 228.746138 (Metazome)

Arthropoda *Daphnia pulex* 341 197.206209

Arthropoda *Ixodes scapularis* 7852 1765.382190

Echinodermata *Strong. purpuratus* 8426 936.564995

Hemichordata *Sacc. kowalevskii* 7194 757.6 (Metazome)

Cephalochordata *B. lanceolatum* 10023 575 (JGI)

Urochordata *C. intestinalis* 6249 115.227500

Cyclostomata *Petromyzon marinus* 1218 885.550958

Cyclostomata *Petromyzon marinus* 2684 885.550958

Chondrichthyes *Callorhincus milli* 11246 1000 (ESGP)

Teleostei *Danio rerio* 14466 1371.719383

Teleostei *Gadus Morhua* 1126 832.114588

Teleostei *Xiphophorus maculatus* 2800 729.664433

Teleostei *Xiphophorus maculatus* 9155 729.664433

Teleostei Takifugu rubripes 818 393.312790

Teleostei Takifugu rubripes 2795 393.312790

Amphibia *Xenopus tropicalis* 8686 1511.735326

Reptiles *Pelodiscus sinensis* 25784 2202.483752

Reptiles *Anolis carolinensis* 26079 1799.143587

Aves *Gallus gallus* 8464 1046.932099

Aves *Ficedula albicollis* 12789 1116.409277

Mammalia *Homo sapiens* 19977 3096.649726

Mammalia *Mus musculus* 8351 2730.871774

Mammalia *Canis lupus fam.* 16520 2410.976875

Mammalia *Ornithorhynchus anat.* 30785 2073.148626

Mammalia *Dasypus novemcinctus* 22513 3631.522711
*unless otherwise stated, source Ensembl – values refer to Golden Path Length.

**Animal group and species** **IG1-intron size (bp)** **DG gene size (bp)****

Porifera *Amphimedon queenslandica* 47 4471

Placozoa *Trichoplax adhaerens* 320 3902

Cnidaria *Hydra magnipapillata* 1751 4270 (Metazome)

Cnidaria *Nematostella vectensis* 2567 8618 (Ens/JGI)

Nematoda *Caenorhabditis remanei* 319 2419

Nematoda *Caenorhabditis elegans* 328 8453

Annelida *Capitella teleta* 48 3666

Annelida *Helobdella robusta* 276 3064

Mollusca *Lottia gigantea* 3043 5365

Mollusca *Crassostrea gigas* 3133 5650

Mollusca *Octopus bimaculoides* 8418 27438 (Metazome)

Arthropoda*Drosophila melanogaster* 284 17389

Arthropoda *Tribolium Castaneum* 46 3762 (Metazome)

Arthropoda *Daphnia pulex* 341 4512

Arthropoda *Ixodes scapularis* 7852 29739

Echinodermata *Strong. purpuratus* 8426 11099

Hemichordata *Sacc. kowalevskii* 7194 18329 (Metazome)

Cephalochordata *B. lanceolatum* 10023 12384 (JGI)

Urochordata *C. intestinalis* 6249 10300

Cyclostomata *Petromyzon marinus* 1218 28315

Cyclostomata *Petromyzon marinus* 2684 5300

Chondrichthyes *Callorhincus milli* 11246 13895 (ESGP)

Teleostei *Danio rerio* 14466 57831

Teleostei *Gadus Morhua* 1126 3781

Teleostei *Xiphophorus maculatus* 2800 16637

Teleostei *Xiphophorus maculatus* 9155 12356

Teleostei Takifugu rubripes 818 3491

Teleostei Takifugu rubripes 2795 5498

Amphibia *Xenopus tropicalis* 8686 31510

Reptiles *Pelodiscus sinensis* 25784 32957

Reptiles *Anolis carolinensis* 26079 90355

Aves *Gallus gallus* 8464 11190

Aves *Ficedula albicollis* 12789 50805

Mammalia *Homo sapiens* 19977 65454

Mammalia *Mus musculus* 8351 57964

Mammalia *Canis lupus fam.* 16520 71092

Mammalia *Ornithorhynchus anat.* 30785 95403

Mammalia *Dasypus novemcinctus* 22513 87666 **unless otherwise stated, source Ensembl.
